# Predictors of Changes in Alcohol Craving Levels during a Virtual Reality Cue Exposure Treatment among Patients with Alcohol Use Disorder

**DOI:** 10.3390/jcm9093018

**Published:** 2020-09-18

**Authors:** Olga Hernández-Serrano, Alexandra Ghiţă, Natàlia Figueras-Puigderrajols, Jolanda Fernández-Ruiz, Miquel Monras, Lluïsa Ortega, Silvia Mondon, Lidia Teixidor, Antoni Gual, Lidia Ugas-Ballester, Maribel Fernández, Roger Montserrat, Bruno Porras-Garcia, Marta Ferrer-Garcia, José Gutiérrez-Maldonado

**Affiliations:** 1 Department of Physical Therapy, Escola Universitària de la Salut i l’Esport (EUSES)-University of Girona, Carrer Francesc Macià, 65, Campus of Salt, 17190 Girona PC, Spain; olga.hernandez@cadscrits.udg.edu; 2 Department of Clinical Psychology and Psychobiology, Faculty of Psychology, University of Barcelona, Passeig de Vall d’Hebron, 175, 08035 Barcelona PC, Spain; alexandraghita@ub.edu (A.G.); nfiguepu11@alumnes.ub.edu (N.F.-P.); jolandafruiz@gmail.com (J.F.-R.); lugas@copc.cat (L.U.-B.); maribelfernandezav@gmail.com (M.F.); rugi_lpps@hotmail.es (R.M.); brporras@ub.edu (B.P.-G.); martaferrerg@ub.edu (M.F.-G.); 3 Addictive Behaviors Unit, Hospital Clinic of Barcelona, Carrer de Villarroel, 170, 08036 Barcelona PC, Spain; mmonras@clinic.cat (M.M.); llortega@clinic.cat (L.O.); smondon@clinic.cat (S.M.); lteixido@clinic.cat (L.T.); tgual@clinic.cat (A.G.)

**Keywords:** alcohol use disorder, craving, virtual reality, cue exposure therapy, treatment-as-usual

## Abstract

Background/Objective: Determining the predictive variables associated with levels of alcohol craving can ease the identification of patients who can benefit from treatments. This study aimed to describe changes (improvement or no change/deterioration) in alcohol craving levels and explore the predictors of these changes from admission to discharge in outpatients with alcohol use disorder (AUD) undergoing treatment-as-usual (TAU), or treatment-as-usual supplemented with virtual reality cue-exposure therapy (TAU + VR-CET). Method: A prospective cohort study was conducted amongst 42 outpatients with AUD (n = 15 TAU + VR-CET and n = 27 TAU) from a clinical setting. Changes in the levels of alcohol craving between admission and discharge were assessed with the Multidimensional Alcohol Craving Scale. Sociodemographic characteristics (age, gender, education, and socioeconomic and civil status), cognitive-affective behavioral patterns (AUD severity, abstinence duration, psychiatric comorbidity, state anxiety, attentional bias, and substance use), and type of treatment (TAU + VR-CET and only TAU) were also evaluated. Results: The TAU + VR-CET group showed greater changes of improvement in the levels of alcohol craving than the TAU group (χ^2^ = 10.996; *p* = 0.001). Intragroup changes in alcohol craving from pre to post-treatment were significant in the TAU + VR-CET group (χ^2^ = 13.818; *p* = 0.003) but not within the TAU group (χ^2^ = 2.349; *p* = 0.503). The odds of an improvement in any of the craving levels between pre- and post-test was 18.18 (1/0.055) times higher in the TAU + VR-CET group with respect to the TAU group. The use of illicit drugs in the month prior to the test increased the odds of having a positive change by 18.18 (1/0.055) with respect to not having consumed. Conclusions: Including VR-CET in TAU programs may provide benefits in the treatment of AUDs mainly among patients with intense alcohol craving and individuals having used illicit substances prior to treatment.

## 1. Introduction

Craving is a multidimensional phenomenon involving an intense urge to consume substances. It is perceived as an individual experience of “wanting” a drug that may result in motivational and drug-seeking behavioral patterns [1,2]. Alcohol craving has been extensively studied due to its clinical implications in the development and maintenance of alcohol use disorder (AUD) [3,4]. Craving is considered one of the mechanisms that promote relapse after treatment discharge [5,6] and even after a prolonged period of abstinence [7]. To better understand the magnitude of the relationship between craving and relapse, it is fundamental to explore the individual variables associated with alcohol craving as it may help in the development of more efficient treatments and strategies to prevent relapse in AUD patients.

Among the factors associated with alcohol cravings are sociodemographic features, such as age, gender, education, socioeconomic status, and civil status, as well as cognitive-affective behavioral patterns, such as attentional bias, active smoking patterns, use of illicit drugs, co-occurring mental health symptoms, psychiatric comorbidities, abstinence, and AUD severity. The influence of *age* in the field of addictive behaviors has been extensively studied. For example, a study indicated that an early drinking age increases the risk of developing a harmful use of alcohol, including the development of AUD, thus highlighting the importance of the age at onset of drinking-related behaviors [8]. Interestingly, another study concluded that alcohol craving decreases with age [9], possibly due to self-regulatory processes and problem-solving abilities in later stages of life improving resilience and coping skills [10]. Regarding *gender* in AUD, the findings from studies are mixed. One study indicated that women experience greater and more prolonged alcohol cravings than men during detoxification [11]. By contrast, an earlier study found no gender differences in alcohol cravings triggered by a cue-exposure paradigm [12]. Another study showed higher levels of alcohol craving reported by women compared to men in a naturalistic bar environment, with the authors of the study concluding that women were at greater risk of continued alcohol use [13]. In terms of *education*, a recent study reported that a higher educational attainment may be associated with a lower risk of alcohol-related behavioral patterns. The study showed that greater educational training interfered with the future development of AUD [14]. However, there is insufficient evidence confirming the direct influence of education on alcohol cravings, with the focus instead being on the negative effects of problematic drinking on students’ academic performances [15]. Similarly, the empirical focus is on the relationship between *socioeconomic/civil status* and alcohol and drug use rather than alcohol craving. Specifically, having a low income and being single predict alcohol and drug use [16].

In terms of the cognitive-affective behavioral mechanisms associated with alcohol craving, studies have shown that implicit cognitive processing, i.e., *attentional bias*, for alcohol-related content predict increased alcohol cravings [17,18]. In relation to *psychiatric comorbidities*, depressive and manic mood states [19], current comorbid mood and anxiety disorders [20], borderline personality disorder [21], or post-traumatic stress disorder [22] have been associated with alcohol cravings. Furthermore, ecological momentary assessment (EMA) studies have found alcohol cravings to be higher after *smoking* [23,24]. In line with this, higher levels of alcohol craving were reported among individuals with active smoking patterns in a residential treatment cohort [25], as well as in individuals who simultaneously use alcohol and *illicit drugs* [26]. Conversely, *abstinence duration* has been reported to be associated with reduced alcohol cravings, suggesting that alcohol cessation as a result of treatment may help to regulate cravings [27]. Several studies have indicated that alcohol cravings predict abstinence and, implicitly, relapse, but less attention has been paid to the predictive role of abstinence in alcohol cravings [5,28,29]. Some studies have found alcohol craving to correlate positively with the *severity of AUD* [30,31,32,33,34], whereas others have not observed such a relationship [35]. In terms of co-occurring mental health symptoms, several studies have demonstrated a causal relationship between psychopathological symptoms and alcohol cravings. For instance, a network modeling analysis emphasized that *anxiety* and stress are directly involved in triggering cravings in AUD patients [36]. These results are supported by those of a previous study, confirming that clinical anxiety and stress-related symptoms are robust predictors of alcohol cravings [37].

There are different approaches in the literature that have been applied to explore alcohol cravings, including the use of neurobiological and psychophysiological correlates [7,38], paper and pencil instruments [39], cognitive/behavioral tasks [18,40], or the cue-exposure paradigm [41]. Cue-exposure techniques are generally used to assess alcohol cravings and are based on in vivo, imagery or virtual exposure. This type of assessment method has been developed into a therapeutic approach: cue-exposure therapy (CET). The theoretical baseline of the CET approach relies on the principles of classical conditioning [42]. Transferred to AUD, alcohol-related stimuli become “highly sensitive stimuli”, as a result of systematic and repetitive alcohol consumption accompanied by positive and rewarding properties of alcohol use. The CET approach, also known as Exposure and Response Prevention (ERP), emphasizes the core mechanisms of systematic desensitization [43]. More specifically, CET/ERP involves repeated and prolonged exposure to alcohol-related stimuli, but individuals do not conduct any drinking behaviors. It is hypothesized that repetitive and systematic exposure will reduce the psychophysiological responses to alcohol-related stimuli, as the ultimate goal of this therapeutic approach is to extinguish initially conditioned responses to alcohol cues (e.g., craving) [44]. Nevertheless, there were inconsistent results regarding the effects of CET approach applied in AUD, mainly because therapy sessions involved exposure to only one cue at a time, within clinical settings, which clearly interferes with generalizing therapy effects into daily-life situations of individuals with AUD. This is one of the major limitations of the CET approach [42].

The rise of technologies like virtual reality (VR) for the past two decades has allowed to complement and upgrade assessment and treatment instruments used in mental health settings. VR has been implemented as a tool to explore and treat different underlying mechanisms and conditions in individuals with mental health disorders [45,46,47,48]. A systematic review encompassing studies using VR as an assessment and treatment tool in individuals who misuse alcohol indicated that VR can be provided with a two-folded purpose: on the one hand, VR-alcohol related environments can elicit significant levels of alcohol craving in individuals with a problematic drinking pattern, and on the other hand, VR can add effectiveness to CET approach (VR-CET) due to its technical features [45]. Compared to traditional CET involving in-vivo or imagery exposure, VR can enhance the efficacy of CET through its multiple sensory inputs (e.g., visual, auditory, olfactory), and ultimately the clinician has a higher control over the exposure process [49]. In addition, VR facilitates simulations of real-life scenarios (including social interaction); it allows a high user-platform interaction; it can provide a fully immersive experience, and therefore, VR can enhance the sense of presence within the exposure [50]. The “sense of presence” is understood as a subjective perception of fully “being” within the VR environment, experiencing the VR scenario as if the individual is in a naturalistic environment [41]. As a result, the individual can better generalize therapy effects and can build coping strategies to further implementing them daily in common situations related to alcohol consumption [45]. For instance, in individuals with AUD, several studies used VR as a therapy tool, emphasizing the VR-CET approach. There were consistent results across these studies, with a great reduction in levels of alcohol craving at post-therapy assessment sessions [51,52,53]. We followed a similar procedure in terms of software development, VR-CET approach, or number of therapy sessions [54].

The main objective of the current study was to determine the predictive relationship between specific variables and alcohol craving in individuals diagnosed with AUD. More specifically, the aims of this study were to (1) describe the changes (improvement or no change/deterioration) in the levels of alcohol craving from admission to discharge during TAU -treatment as usual- + VR-CET and only TAU and (2) examine whether sociodemographic characteristics, cognitive-affective behavioral patterns, and the type of treatment (TAU +VR-CET or TAU) predicted changes in the levels of alcohol craving from admission to discharge during treatments in outpatients diagnosed with AUD. 

## 2. Methods

### 2.1. Participants

**Figure 1:**
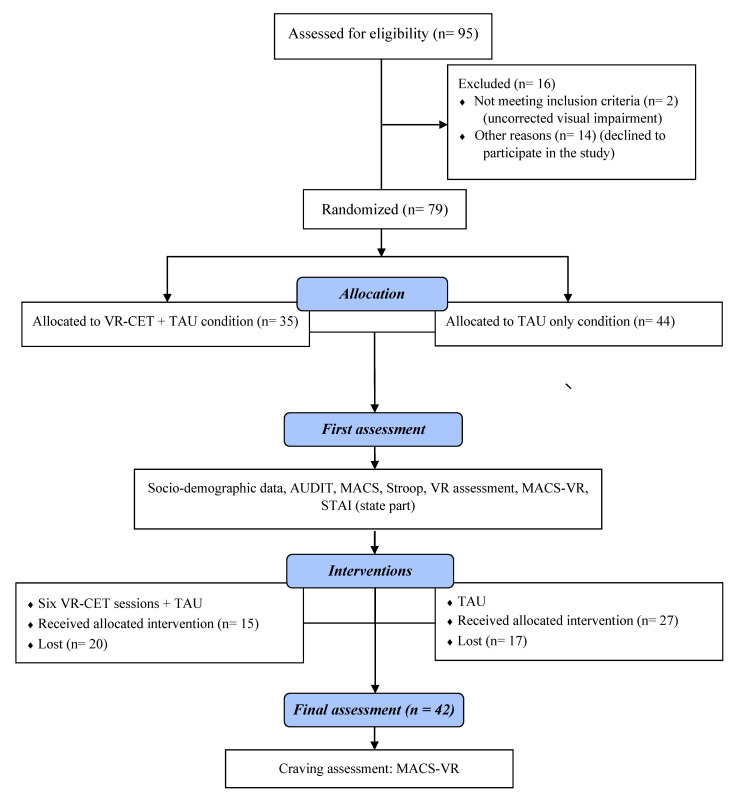
Flowchart of the study. AUD = alcohol use disorder; AUDIT = Alcohol Use Disorders Identification Test; MACS-VR = Multidimensional Alcohol Craving Scale—Virtual Reality; STAI = State-Trait Anxiety Inventory; TAU = treatment–as-usual; VR = virtual reality; VR-CET = virtual reality cue-exposure therapy.

A total of 95 participants were identified during the study period. Of these, 16 either did not fulfill inclusion criteria or were excluded for other reasons (e.g., uncorrected visual impairment or work commitments clashed with the treatment schedules). All of them were recruited from the Hospital Clinic of Barcelona. The inclusion criteria were an AUD diagnosis according to the criteria of the *Diagnostic and Statistical Manual of Mental Disorders* (5th Ed.) [55] and outpatient TAU for AUD at the Hospital Clinic of Barcelona. In addition, we focused on patients who were considered “resistant-to-TAU”, meaning that they had experienced relapse in the first six months after treatment discharge from the Addictive Behaviors Unit of the Hospital Clinic of Barcelona. The lead clinical psychologist from the hospital selected these patients based on their clinical history while the patients were under ambulatory treatment (receiving TAU) at the moment of this study. This outpatient treatment includes pharmacotherapy and psychosocial care. The exclusion criteria were severe psychopathology (e.g., psychosis), severe cognitive impairment, use of anti-craving medication (e.g., naltrexone), and pregnancy. All patients meeting the inclusion criteria were then consecutively and randomly assigned to one of the two experimental conditions described below. Thus, 79 AUD patients, aged 36 to 67 years, were enrolled into the study (35 patients were assigned to the ”VR-CET + TAU” group and 44 were assigned to “TAU” only). Of these, 37 participants did not complete all treatment sessions, and 42 participants received the assigned intervention and the pre and post-test evaluation: 15 VR-CET + TAU and 27 only TAU (see Figure 1). The ethical code number is 0377 (HCB/2017/0377) and the approval date was 09/2017.

### 2.2. Measures

*Alcohol consumption, drinking behaviors, and alcohol-related problems* were screened using the Alcohol Use Disorders Identification Test (AUDIT) [56]. The Spanish version of AUDIT is a 10-item self-report tool that aims to detect excessive alcohol consumption. This version is also used as a support to clinicians in brief evaluations [57]. It includes three questions on the consumption of alcoholic drinks (amount and frequency), four questions related to dependence, and three questions on the analysis of its consequences. The questions are scored from 0 to 4 points, except questions 9 and 10, which are scored as 0, 2, or 4 points. The final score ranges from 0 to 40 points. A total score equal to or higher than 8 points indicates hazardous drinking and harmful consumption as well as possible alcohol dependence. In this study, the AUDIT score was used as an indicator of AUD severity [58].

*Alcohol craving* was explored by means of an ad-hoc modified version of the Multidimensional Alcohol Craving Scale (MACS), to explore alcohol cravings immediately after VR exposure. MACS [59] is a self-report scale aiming to determine the “intensity of alcohol craving experienced by the participant in his/her previous week”. This scale included two sub-scales, “desire to drink” and “behavioral disinhibition”. The MACS has 12 items scoring from 1 (“strongly disagree”) to 5 (“strongly agree”). Thus, it classifies a craving as non-existent (0–12), mild (13–22), moderate (23–40), or intense (>40). The modified version of the MACS used in this study (MACS-VR) requests participants to determine the “intensity of alcohol craving experienced during VR exposure”, with the scores remaining the same as those of the original version, including the “desire to drink” and behavioral disinhibition” sub-scales. The total craving score was calculated by adding up the results from the “desire to drink” and “behavioral disinhibition” sub-scales.

*Attentional bias* for alcohol-related content was assessed using the Spanish version of the Alcohol Stroop task [60]. This task aims to determine the interference of alcohol stimuli in AUD and consists of three parts, with alcohol-related words presented in black and white or in color. The words in each part must be read out as fast as possible over 45 s. Final scores of the Stroop task indicate the level of attentional bias towards alcohol-related stimuli.

*State anxiety* was explored with the Spanish version of the State-Trait Anxiety Inventory (STAI) [61], which is a self-report questionnaire assessing how a person is feeling at the moment (STAI-State, State Anxiety). The scale consists of 20 items, which are scored from 0 (“not at all”) to 3 (“very much so”). Higher scores of each subscale indicate higher levels of state anxiety.

### 2.3. Instruments

#### 2.3.1. Hardware

The VR equipment consisted of an Oculus Rift S head-mounted display (HMD) (Oculus VR, Irvine, CA, USA), sensors, touch controllers and a computer compatible with VR technology.

#### 2.3.2. Software

**Figure 2:**
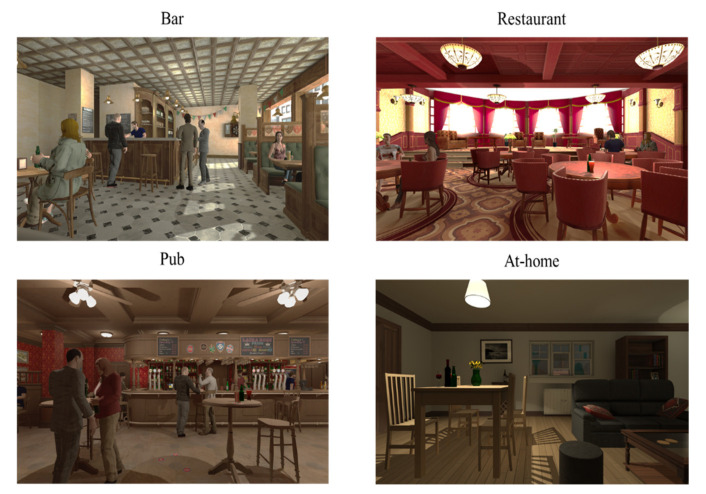
Images of the four alcohol-related VR environments.

The ALCO-VR software (developed by the VR-Psy Lab, University of Barcelona, Spain) is a VR platform that was implemented in the current study for the assessment and treatment of AUD patients. The software was developed based on the outcomes of previous studies conducted at the University of Barcelona. The first of these studies, investigating the triggering factors of alcohol cravings in AUD patients, found that the cues and contexts that most elicited cravings were a restaurant, a bar, a pub, and home environments. Thus, VR environments were created that were based on the realistic scenarios reported by AUD patients. The four VR environments created included social interactions (human avatars), a wide variety of alcoholic beverages (bottles of alcohol were displayed in the backgrounds of the VR environments), and different times of the day (daytime or night time) [62]. Figure 2 presents screenshots of these four VR environments. A major objective of the software was to allow a high level of interaction between the participant and the simulation, which is fundamental in augmenting the sense of presence within VR environments. Therefore, the VR setup produced realistic movement of the wrists and feedback, allowing the participant to approach alcoholic drinks and other objects within the VR environment. Another study, assessing the VR platform in terms of cravings and anxiety in a sample of AUD patients and social drinkers (SD), indicated that the VR software was an ecologically valid instrument, as AUD patients reported significantly higher levels of cravings and anxiety than SD [63].

The software consisted of two parts, assessment and therapy. The assessment part aimed to establish a hierarchy of exposure from the lowest-rated environment with the lowest-rated alcoholic drink to the highest-rated environment and the highest-rated alcoholic drink. Considering the perceived momentary alcohol cravings, the participants were asked to rate environments and alcoholic beverages on the VAS from 0 to 100, 0 being “no alcohol craving” and 100 being “intense alcohol craving”. Thus, the platform selected the top five rated alcoholic beverages, which were the most preferred drinks of each participant. The therapy part of the software was based on the data provided in the assessment part and consisted of prolonged exposure to the cues and contexts selected previously by the user.

### 2.4. Procedure

Ethical approval for the study was obtained from the ethics committees of both the University of Barcelona and the Hospital Clinic of Barcelona. AUD patients from the Addictive Behaviors Unit of the Hospital Clinic of Barcelona were invited to participate in the study after they had provided their written informed consent. All patients were administered an initial assessment session to explore underlying mechanisms of AUD, which was repeated at four to five weeks, depending on the availability of each patient. Patients assigned to TAU condition received only these two assessment sessions. The protocol for patients assigned to VR-CET + TAU consisted of eight sessions: an initial assessment, six VR-CET sessions, and a final assessment, in addition to their baseline TAU at the hospital. The initial assessment for all patients consisted of collecting sociodemographic data of the patients, including information on their AUD history, use of substances other than alcohol, dual diagnosis, and abstinence. In this session, patients completed the AUDIT, STAI-State, and Alcohol Stroop Task, as well as the VR assessment task followed by the completion of the MACS-VR. The VR assessment task first involved familiarizing the participants to the VR technology through a short tutorial prior to the assessment itself, using the HMD and controllers. The patients then reported their momentary craving levels when exposed to alcoholic drinks and alcohol-related environments on VAS (0–100). The software recorded the data and created a hierarchy based on each patient’s preferred alcoholic drinks and alcohol-related environments. The exposure time was 20 s in each environment and for each drink. This phase was fundamental as the hierarchy was further used in the therapy part. To increase realism during VR exposure, olfactory stimuli were introduced into the protocol. A small amount of the alcoholic beverage was transferred onto cotton pads and placed close to each participant, corresponding to the alcoholic drinks being displayed in the VR platform. The VR assessment task lasted approximately 10–15 min. After the task, patients completed the MACS-VR. The initial assessment session lasted approximately one hour.

Then patients assigned to the VR-CET + TAU group were scheduled their first therapy session, whereas patients in the TAU group were informed that a final assessment session would be conducted after 4–5 weeks. All patients (VR-CET + TAU and TAU only) kept their baseline treatment (TAU) at the Hospital Clinic of Barcelona. The final assessment session was administered three days after the completion of the VR-CET intervention in the VR-CET group. The post-therapy evaluation consisted of completing the MACS-VR after cue-elicited craving using VR exposure to alcohol-related contexts and cues. We used the “assessment” part of the ALCO-VR software for the initial and final evaluation sessions, whereas for the VR-CET sessions we used the “therapy” part of the same software. The ratings of momentary levels of alcohol craving during assessment and therapy sessions were not included in the current study as this study is part of an ongoing clinical trial.

The entire experimental protocol for each patient lasted approximately five weeks, including both assessments and therapy. The VR-CET approach was delivered by experienced practitioners. Patients received short debriefings at the end of the sessions. Patients were allowed to disclose any thoughts, emotions or behaviors related to further alcohol consumption, and were counseled by the experimenter until craving levels were minimal. The debriefing intervention was facilitated to prevent any possible consumption.

#### 2.4.1. Randomization

This study is part of an ongoing clinical trial testing the efficacy of the VR-CET + TAU approach versus TAU only in resistant-to-TAU AUD individuals. While the emphasis of the current study does not rely on the results of the clinical trial itself, we highlighted the predicting role of individual variables in determining alcohol craving in AUD patients. The project reflects a single-blinded randomized trial, where the participants were not informed whether they had received the “control” (TAU only) or “experimental” (TAU + VR-CET) condition. We randomly assigned the participants to one of the groups by following one of the common methods of simple randomization, flipping a coin [63]. Before the participants were scheduled to attend the first assessment session, the experimenter flipped a coin to match the new patient to one of the conditions.

#### 2.4.2. VR-CET Condition

Patients assigned to the experimental condition were administered eight sessions (two assessment sessions and six therapy sessions). Patients in this group received the six VR-CET sessions while they were continuing with their baseline treatment (TAU) at the hospital. Therefore, the treatment protocol in this group emphasized the “therapy” part of the “ALCO-VR” software. This protocol included *only* cue-exposure therapy (exposure and response prevention), to underline the core mechanisms of systematic desensitization, meaning systematic and prolonged exposure to the same preferred five alcohol-related stimuli in the four VR environments. There were no other psychotherapeutic methods included during VR-CET sessions. As there was a high level of user-simulation interaction, instructions were given to the patients to grab the alcoholic drink, observe it from all the angles, but without attempting to “virtually” drink from it. Such approach highlights the habituation process through response prevention. Similar to the assessments part, we included olfactory stimuli in all therapy sessions, with alcoholic beverages placed on cotton pads corresponding to the drinks explored during VR exposure. All patients underwent VR-CET sessions twice a week over the course of three weeks. Each VR-CET session lasted 50 min.

The therapy sessions were based on the outcomes of the initial assessment session, and the exposure involved interplay between the first five preferred alcoholic beverages within each one of the four VR environments. The four VR alcohol environments were always the same: pub, bar, restaurant, and at-home. Only the five alcoholic beverages changed according to the hierarchy of the software. The first VR-CET session started with the lowest rated alcoholic drink (from the first five preferred alcoholic beverages) within the lowest rated alcohol-related VR environment. The VR-CET approach involved gradual exposure from the *lowest rated* to the *highest rated* alcoholic beverages and environments. The software allowed the participants to continue to the next level only if they scored 40% less, three times in a row, than their initial rating for cravings. “Next level” represented a new alcoholic beverage from the first five preferred ones (combined with the same four VR environments) that was rated higher in the gradual hierarchy. If the ratings were not sufficiently lower in order to continue to the next alcoholic drink, the patient remained within the same VR environment with the same alcoholic beverage. Every 60 s, patients were asked to rate their momentary levels of alcohol craving. The same procedure was followed for all of the six VR-CET sessions. If patients had completed all the levels (all five preferred alcoholic drinks within each one of the four VR environments), they started again the hierarchy. In our study, there was variability in terms of completing the VR-CET protocol, with some patients being exposed to the same alcoholic drink within the same VR environment for one entire therapy session, while there were other patients completing all levels twice.

#### 2.4.3. TAU Condition

Participants assigned to either TAU only or TAU + VR-CET conditions received the same baseline treatment, that is, classical treatment (TAU) at Hospital Clinic of Barcelona. All participants received therapy on an individual and group basis. The primary therapeutic goal of TAU was to maintain abstinence from alcohol during and after treatment discharge. The treatment consisted of a combination of pharmacotherapy and psychotherapy. Pharmacotherapy generally included medication such as disulfiram, anxiolytics, and/or antidepressants. Individual and group therapy sessions were based on psychotherapeutic approaches like cognitive-behavioral therapy and motivational interviewing. Weekly groups included facilitated recovery-oriented discussions in an open-group format. The TAU groups met once or twice weekly for 1½ hours. Patients assigned to the TAU condition were administered two assessment sessions with a 4–5-week difference in-between.

### 2.5. Data Analysis

Descriptive statistics were used to characterize the participants at baseline. Baseline differences between the two treatment groups (TAU vs. TAU + VR-CET) were also assessed through Student t-Test or Chi-squared test when suitable. Change was assessed by comparing the levels of alcohol craving before and after the intervention. The results were categorized into 2 patterns: improvement and no change/deterioration. In addition, participants showing improvements were grouped based on the levels of improvement (e.g., one level, where participants improved from having an “intense” alcohol craving to a “moderate” craving; two levels, where participants improved from having an “intense” alcohol craving to a “mild” craving; and three levels, where participants improved from having an “intense” alcohol craving to a “non-existent” craving).

To assess whether there was a significant reduction in craving from pre to post-treatment, Chi-squared tests were conducted for the entire sample and also within groups of treatment. Post-test differences between the two treatment groups (TAU vs. TAU + VR-CET) were also assessed through Chi-squared test.

Chi-squared test or Fisher’s exact test and independent t-test were used to determine the relationships between predictors and categorical and quantitative variables, respectively; associations were tested for a significant relationship (*p* < 0.05; [64]) with the change in alcohol craving levels. In binary logistic regression analysis, the factors with statistically significant contributions concerning the outcome measure (changes in alcohol craving levels) were combined and used as independent variables, with “change” (improvement = 1; no change or deterioration = 0) as the dependent variable. The final model was produced with the enter method. Analyses were performed using SPSS version 24.0 (IBM Corp., Armonk, NY, USA).

## 3. Results

The characteristics of the participants at baseline are described in Table 1. In total, 42 AUD patients (50.0% females) with a mean age of 54.6 years (SD = 7.71) completed the intervention. Among the participants, 40.5% had a high school education, while a high percentage of the patients had a medium socioeconomic status (83.3%). Most patients were married/in a relationship (45.2%) or separated/divorced (31.0%). In our sample, 52.4% showed psychiatric comorbidities, with depression being the most common (28.6%). In addition, 59.5% and 35.7% of the participants were tobacco and illicit drug users, respectively. The mean abstinence period at baseline was three months and the average score for AUDIT was 16.95.

At baseline, and concerning the TAU + VR-CET group, 4 patients had no craving, 10 patients had mild level of craving, whereas 8 showed moderate and 5 had intense levels of craving. As for the TAU group, 0 patients had no craving, whilst 7 displayed mild level, 3 had moderate and 5 showed intense levels of craving. Both groups showed no significant differences at baseline (χ^2^ = 3.673; *p* = 0.299).

**Figure 3:**
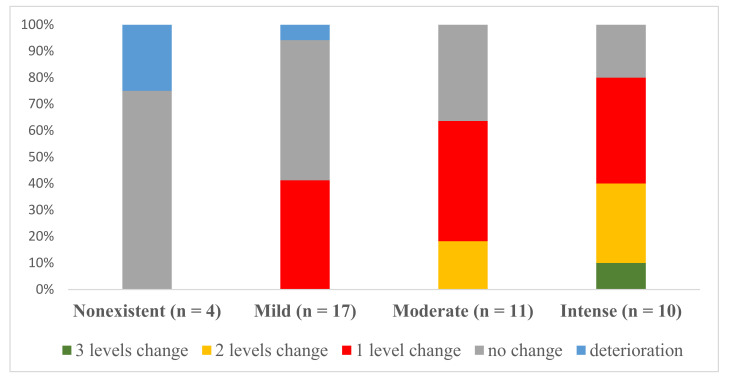
Proportion of the sample exhibiting patterns of change after treatment for the different levels of alcohol craving at admission in the whole sample (TAU + VR-CET and TAU) (n = 42).

Figure 3 displays changes in the levels of alcohol craving for each one of the levels at admission in the whole sample (n = 42). It shows the proportion of participants whose craving level did not change/deterioration (i.e., from “moderate” to “moderate” or “moderate” to “intense”), those who showed an improvement of one level (i.e., from “intense” to “moderate” for instance), those who showed an improvement of two levels (i.e., from “intense” to “mild” for example), and those who showed an improvement of three levels (i.e., from “intense” to “non-existent”, for example). Participants with mild or non-existent alcohol cravings at admission displayed a lower level of change. By contrast, those with intense or moderate alcohol cravings demonstrated a greater level of change, with a higher proportion of participants improving by one level. At the end of the treatment, 52.4% of the participants showed improvements (16 (38.1%) improved by one level, 5 (11.9%) improved by two levels, and 1 (2.4%) improved by three levels); 18 (42.9%) presented no change; and 2 (4.8%) deteriorated). Consequently, at discharge, 2 (4.8%) of the participants had intense alcohol cravings, 13 (31.0%) showed non-existent cravings, 9 (21.4%) had a moderate level of alcohol craving, while 18 (42.9%) reported mild cravings.

**Figure 4:**
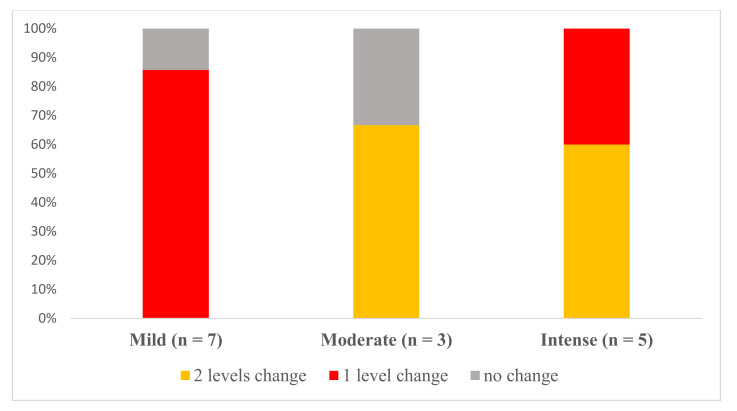
Proportion of the sample exhibiting patterns of change after treatment for the different levels of alcohol craving at admission in the TAU + VR-CET group (n = 15).

Figure 4 displays changes in the levels of alcohol craving for each one of the levels at admission in the TAU + VR-CET group. Participants with moderate alcohol cravings at admission displayed a lower percentage of change after treatment. By contrast, those with intense alcohol cravings demonstrated a greater level of change, with a higher proportion of participants improving by one and two levels. At the end of the treatment, 86.6% of the participants showed improvements (8 (53.3%) improved by one level, 5 (33.3%) improved by two levels), 2 (13.3%) presented no change, and 0.0% deteriorated. Consequently, at discharge, 8 (53.3%) showed non-existent cravings, 3 (20.0%) had a moderate level of alcohol craving, while 4 (26.7%) reported mild cravings.

**Figure 5:**
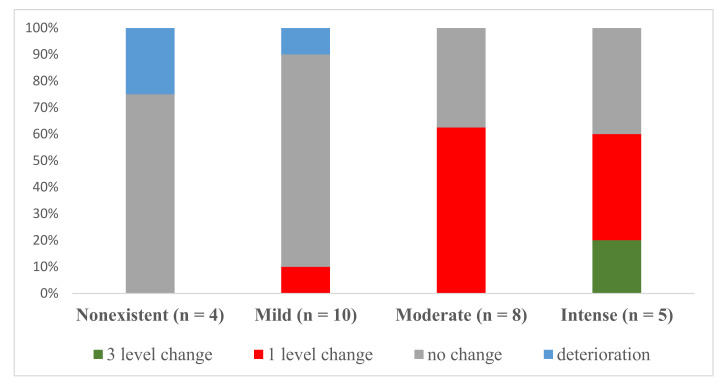
Proportion of the sample exhibiting patterns of change after treatment for the different levels of alcohol craving at admission in the TAU group (n = 27).

Figure 5 displays changes in the levels of alcohol craving for each one of the levels at admission in the TAU group. Participants with mild alcohol cravings at admission demonstrated a lower level of change after treatment. However, those with moderate alcohol cravings demonstrated a greater level of change, improving by one level. At the end of the treatment, 33.3% of the participants showed improvements [8 (29.6%) of the participants showed improvements by one level and 1 (3.7%) participant showed improvement by three levels], 16 (59.3%) presented no change, and 2 (7.4%) deteriorated. Consequently, at discharge, 2 (7.4%) of the participants had intense alcohol cravings, 5 (18.5%) showed non-existent cravings, 6 (22.2%) had a moderate level of alcohol craving, while 14 (51.9%) reported mild cravings.

Changes in craving from pre to post-treatment were significant, stating significant reduction in craving amongst the entire sample (χ^2^ = 10.326; *p* = 0.015) and within the TAU + VR-CET group (χ^2^ = 13.818; *p* = 0.003) but not within the TAU group (χ^2^ = 2.349; *p* = 0.503).

At post-test, and concerning the TAU + VR-CET group, 13 patients showed improvement changes whilst 2 patients showed no change or deterioration. As for the TAU group, 9 patients showed improvement changes, whereas 18 patients did not change or deteriorated. Both groups showed significant differences at post-test (χ^2^ = 10.996; *p* = 0.001).

No statistically significant differences were found concerning the sociodemographic characteristics (age, gender, education, and socioeconomic status), abstinence duration, psychiatric comorbidity, state anxiety, and attentional bias (Table 1). These analyses provide a relative measure of the odds of experiencing an improvement or no change/deterioration in the levels of alcohol craving among the participants. Based on the significant relationships (*p* < 0.05), as stated elsewhere [64], two factors were entered into a logistic regression model: type of treatment (TAU + VR-CET or TAU) and the use of illicit substances in the month prior to the baseline.

Table 2 presents the results from the binary logistic regression analysis. The odds of an improvement in any of the craving levels between pre- and post-test were 18.18 (1/0.055) times higher within the TAU + VR-CET group with respect to the TAU group, indicating that TAU + VR-CET group had a positive impact on the odds of experiencing an improvement. The use of illicit drugs in the month prior to the test increased the odds of having a positive change by 18.18 (1/0.055) with respect to not having consumed. The model fitted well (χ^2^ = 23.301; *p* < 0.001) and explained more than 50% of the variability (Nagelkerke’s R^2^ = 0.568).

## 4. Discussion

The main objectives of this study were to (1) describe the changes (improvement or no change/deterioration) in the levels of alcohol craving from admission to discharge during TAU + VR-CET and only TAU and (2) examine whether sociodemographic characteristics, cognitive-affective behavioral patterns, and the type of treatment (TAU +VR-CET or TAU) predicted changes in the levels of alcohol craving from admission to discharge during treatments in outpatients diagnosed with AUD. In line with some previous studies [9,12,15,16,35], sociodemographic characteristics and some cognitive-affective behavioral variables were not predictors of change in alcohol craving levels. However, the type of treatment and the use of other substances within the month prior to treatment were associated with improvements in the levels of alcohol craving after treatments. Furthermore, the TAU + VR-CET group showed greater changes of improvement in the levels of alcohol craving than the TAU group.

Among all the participants (n = 42), more than half of them (52.4%) showed improvements in their initial level of alcohol craving. The highest proportion of participants showing a change were those who improved by one level. The number of participants with moderate or intense cravings at discharge was lower than that reported on the pre-assessment. Similar results were found in two previous studies, which reported that the best responses to the anti-craving agent naltrexone were associated with high alcohol craving scores [65,66]. A possible explanation for our results could be negative alcohol expectancies. For example, AUD patients with long-term negative expectancies about alcohol may regulate alcohol cravings the best. This explanation is supported by a study that tested whether AUD patients could successfully recruit the prefrontal cortex to effectively regulate cravings and found that AUD patients could use cognitive strategies to reduce cue-induced alcohol craving, with those reporting greater long-term negative alcohol expectancies being more successful at regulating their cravings [67]. Further studies should investigate whether AUD patients with moderate or intense cravings and with negative expectancies on alcohol are more efficient in regulating their alcohol cravings.

Our results indicated that AUD patients with intense or moderate cravings at admission formed only a subgroup of all patients with AUD (50.0%). In other words, not all AUD patients who had made several failed attempts to stop drinking alcohol reported high levels of alcohol craving (mild craving = 40.5% and non-existent craving = 9.5%). A possible explanation is the relationship between impulsivity and alcohol craving. Some authors found that the increase in impulsivity is related to stronger impulses to drink among alcohol dependent patients [68,69]. However, some studies have indicated that patients with AUD have a tendency to avoid or minimize their condition [70]. Our results could also be explained by the abstinence period since this ranged from 4 days to a year in our study participants. This finding is consistent with other studies noting that cravings may be temporary and transient [71,72] and may decrease with prolonged abstinence time. Although cravings could persist for a long period of time, the level of craving will decrease during a prolonged period of remission [73,74].

The type of treatment predicted changes in alcohol craving. The TAU + VR-CET group showed greater changes of improvement in the levels of alcohol craving than the TAU group. Taking into account the levels of change within each group, in the TAU + VR-CET group, participants with intense alcohol cravings demonstrated a greater level of change, with a higher proportion of participants improving by one and two levels. In contrast, in the TAU group, no differences were found in alcohol craving from pre to post-treatment. These findings suggest that including VR-CET in TAU programs may provide benefits in the treatment of AUDs. In this sense, AUD patients with different levels of craving may require different clinical management. For example, TAU + VR-CET treatment could be administered mainly to intense craving patients, instead of all AUD patients. Having a VR experience targeting alcohol craving promotes systematic desensitization to alcohol-related cues and contexts [41]. Such therapeutic approach outweighs traditional CET [42]. The type of treatment may be more individualized depending on the intensity of craving. Future studies with a deeper understanding of the levels of change or the intensity of alcohol craving could lead to more effective interventions for the treatment of AUD patients.

In the present study, AUD patients visualized different virtual environments (a restaurant, a bar, a pub, and home) that included social interactions (human avatars) and different times of the day (daytime or nighttime). A wide variety of alcoholic beverages were used (alcohol bottles were displayed in the backgrounds of the VR environments), but not other types of substances (e.g., cannabis or cocaine). The use of illicit substances within the month prior to treatment was found to be a predictor of reduced alcohol cravings. Considering that frequent or excessive consumers of alcohol are more likely to report cannabis and cocaine use compared to the general population [75,76], this finding could be attributed to the subject’s own expectations [77]. For example, the presence of a virtual scenery strongly linked to alcohol and the use of illicit substances (e.g., virtual scenery linked to co-use of alcohol and cannabis), but without explicit illicit substance cues (i.e., only the alcohol cue), may provoke illicit substances cravings among AUD patients. This is supported by studies showing that a smoking-related scenery without explicit smoking cues may still provoke cravings [78,79].

Our findings should be interpreted in light of some methodological limitations. First, the relatively small sample size of the study makes it difficult to generalize our results to other patients with AUD. Second, the vast majority of studies in the literature use different types of assessment strategies (e.g., signal contingent, event-contingent recordings, or interval contingent), and their results are hardly comparable to those we obtained with the assessment strategy of measuring changes in alcohol craving levels. Similarly, different types of instruments have been used in the literature to measure the severity of dependence (e.g., Structured Clinical Interview for Diagnostic and Statistical Manual Disorders, Fourth or Fifth Edition), which hinders the comparability of the results. Third, the greatest level of change occurred in the VR-CET group: Individuals from this group had higher levels of craving at baseline, and therefore, generalizations on this aspect should be made cautiously. Fourth, the length of the abstinence period in the present study was variable among the AUD patients. In this sense, future studies should consider testing the effectiveness of the ALCO-VR software in discriminating and differentiating between patients with long-term versus short-term abstinence periods in terms of alcohol cravings. Fifth, the present study did not evaluate the co-use of alcohol and other substances; however, alcohol and tobacco followed by illicit drugs (especially cannabis) form a frequent pattern of drug combinations amongst young adults in Europe [75,76]. Several studies have found that the co-use of alcohol and illicit drugs is significantly associated with alcohol cravings [26]. Furthermore, alcohol cravings might vary in patients depending on the frequency or quantity of substance use. Additional studies in AUD patients with concurrent polydrug use prior to treatment, taking into account both frequency and quantity, would help to verify and extend these preliminary results. On the other hand, in our study, 46.83% of AUD patients expressed their intention to drop-out from the ALCO-VR program. Previous research indicated that treatment abandon, commonly known as “drop-out”, is a frequent concern among individuals with substance use disorders (SUD). Drop-out rates vary between 25% and 50% among in-/outpatients with SUD and negatively impact treatment outcomes by facilitating the risk of relapse [80,81]. Hence, our study confirms existing results regarding drop-out rates, and we recommend future research to explore this phenomenon to minimize treatment discontinuation. We aim to examine alcohol relapse once the clinical trial is completed through follow-ups at 3, 9, and 12 months. Finally, as this is an ongoing study, we did not include ratings of momentary levels of alcohol craving during exposure to VR-alcohol related cues and contexts in both assessment and therapy sessions. Once the ongoing clinical trial is completed, the data will be thoroughly analyzed.

## 5. Conclusions

In conclusion, the TAU + VR-CET group showed greater changes of improvement in the levels of alcohol craving than the TAU group. AUD patients with intense alcohol cravings showed greater levels of change within the TAU + VR-CET group. By contrast, within the TAU group, no differences from pre to post-treatment were found in alcohol craving. The type of treatment and the fact of having consumed in the previous month were two factors associated to a greater improvement of alcohol craving. These findings suggest that including VR-CET in TAU programs may provide benefits in the treatment of AUDs mainly among patients with intense alcohol craving and individuals who use illicit substances prior to treatment. Therefore, these predictors of changes in the levels of alcohol craving can be used to tailor therapy. However, as noted, further research with larger samples and follow-up data is needed, as well as inclusion of ratings of momentary levels of alcohol craving during exposure to VR-alcohol related cues and contexts.

## Figures and Tables

**Table 1:** Participant characteristics at admission (n = 42).

	N (%) or Mean ± SD	N or Mean ± SD			Comparison TAU vs. TAU+VR-CET
Characteristics	Total Sample (n = 42)	Improvement in AC (n = 22)	No Change or Deterioration (n = 20)	*p* Value	*p* Value
Age	54.60 ± 7.71	52.91 ± 7.74	56.45 ± 7.42	0.474	0.576
Gender (female)	21 (50.0%)	13	8	0.354	1.000
Education				0.617	0.657
Elementary school	0 (0.0%)	0	0		
High school	17 (40.5%)	9	8		
Junior college Associate degree	9 (21.4%)	6	3		
University	15 (35.1%)	6	9		
Master‘s degree	1 (2.4%)	1	0		
Socioeconomic status				0.449	0.271
Low	6 (14.3%)	4	2		
Medium	35 (83.3%)	17	18		
High	1 (2.4%)	1	0		
Civil status				0.116	0.571
Single	5 (11.9%)	1	4		
Married/in a relationship	19 (45.2%)	8	11		
Separated/divorced	13 (31.0%)	9	4		
Widower	5 (11.9%)	4	1		
AUD severity	16.95 ± 9.96	18.55 ± 11.15	15.20 ± 8.40	0.068	0.572
Abstinence duration (days)	98.52 ± 113.72	93.95 ± 109.96	103.55 ± 120.38	0.975	0.538
Psychiatric comorbidity	22 (52.4%)	11	11	0.746	1.000
Types of psychiatric comorbidity				0.133	0.199
No comorbidity	20 (47.6%)	11	9		
DD	12 (28.6%)	3	9		
DD + AD	2 (4.8%)	1	1		
DD + AD + PD	3 (7.1%)	2	1		
AD	2 (4.8%)	2	0		
PD	3 (7.1%)	3	0		
State anxiety	18.47 ± 11.37	18.40 ± 11.32	18.55 ± 11.72	0.710	0.326
Attentional bias	15.36 ± 13.15	13.36 ± 13.94	17.55 ± 12.20	0.560	0.491
Current smoker	25 (59.5%)	15	10	0.546	0.531
Use of Illicit drugs	15 (35.7%)	13	2	0.001	0.325
Type of treatment				0.001	NA
TAU + VR-CET	15 (35.7%)	18	9		
TAU	27 (64.3%)	2	13		

SD = standard deviation; AC = alcohol craving; AUD = alcohol use disorder; TAU = treatment-as-usual; TAU + VR-CET = treatment-as-usual supplemented with virtual reality cue-exposure therapy; AD = anxiety disorder; PD = personality disorder; DD = depression disorder; NA = Non-applicable.

**Table 2:** Logistic regression analyses, with changes in the levels of alcohol craving as the dependent variable.

Characteristics	B	O.R. (95%CI)	*p* Value
Type of treatment	−2.899	0.055 (0.008–0.386)	0.004 *
Illicit drugs	−2.899	0.055 (0.008–0.386)	0.004 *

NA = not applicable; O.R. = odds ratio; * *p* < 0.05; Ref = reference category; CI = confidence interval.
